# A New Life Satisfaction Scale Predicts Depressive Symptoms in a National Cohort of Older Japanese Adults

**DOI:** 10.3389/fpsyt.2020.00625

**Published:** 2020-07-14

**Authors:** Hiroyuki Shimada, Sangyoon Lee, Seongryu Bae, Ryo Hotta

**Affiliations:** ^1^ Center for Gerontology and Social Science, National Center for Geriatrics and Gerontology, Obu, Japan; ^2^ Department of Early Childhood Education, Kyushu Junior College of Kinki University, Iizuka, Japan

**Keywords:** life satisfaction, depression, Geriatric Depression Scale, older, reliability

## Abstract

**Background:**

Existing satisfaction measures cover general feelings of well-being among older adults, but it is not clear whether life satisfaction is associated with depressive symptoms that decrease psychological well-being. We developed a new life satisfaction scale to assess the associations of health, social factors, and interpersonal relationships with overall life satisfaction. The structural and predictive validity of the scale regarding the onset of depressive symptoms was examined.

**Methods:**

A 13-item questionnaire was developed based on a literature review. The response options for all of the questions were: 1 = poor, 2 = not very good, 3 = good, and 4 = excellent. For the analysis, a total satisfaction score was calculated by summing the individual scores (range = 13–52), and higher scores indicated higher overall satisfaction. Baseline data were obtained using the Geriatric Depression Scale-Short Form with a 30-month follow-up. Older Japanese adults (*n* = 1,792, mean age 70.1 ± 6.3 years, 46.4% male) participated in both surveys. An exploratory factor analysis and a logistic regression analysis were used to verify the construct and predictive validity.

**Results:**

In the exploratory factor analysis, a four-factor structure consisting of Personal Satisfaction, Societal Satisfaction, Community Satisfaction, and Health Satisfaction explained 63% of the total variance, with factor weights between 0.42 and 0.90, and internal reliability was acceptable (Cronbach’s alpha = 0.86). About 13.8% of the sample was identified as having depressive symptoms during the follow-up period. The Youden index determined the cutpoints regarding the development of depressive symptoms identified by the life satisfaction scale as 39 points. The participants were classified as high life satisfaction (60%, *n* = 1,068) or low life satisfaction (40%, *n* = 724). The logistic regression analysis revealed that the scale (cutpoint at 39 points) predicted depressive symptoms while controlling for the effects of other relevant variables (OR = 5.14, 95% Confidence Interval 3.76–7.04, *p* < 0.001). The relative risk of the low life satisfaction group for developing depressive symptoms compared to the high life satisfaction group was 2.39.

**Conclusions:**

The 13-item questionnaire is a valid instrument for measuring the risk of depressive symptoms in older adults.

## Introduction

Japan scores high on some well-being measures in the Better Life Index, and it ranks first on personal security. Japan is above the Organisation for Economic Co-operation and Development (OECD) average for income and wealth, education and skills, employment, housing, personal security, and environmental quality. However, the country is below average regarding civic engagement, subjective well-being, social connections, work-life balance, and health status. In general, Japanese people are less satisfied with their lives than the OECD average. When asked to rate their overall satisfaction with life on a scale from zero to 10, the average Japanese score of 5.9 was lower than the OECD average of 6.5 (1). Life dissatisfaction has been related to a higher prevalence of suicide as well as mental disorders, including depression, in the United States (2), and a seven-year longitudinal study in Finland found that dissatisfaction with life predicted the onset of major depressive disorder (3).

Usually, a simple index is used to measure life satisfaction in clinical and primary care settings. The Satisfaction With Life Scale (SWLS) (4) is heavily relied on to measure the life satisfaction component of subjective well-being. The SWLS has been correlated with measures of depression and positive emotions (5) and is employed to predict various behaviors, such as suicide ideation (6). The SWLS can integrate and weight various dimensions, but it does not assess satisfaction with specific life domains, such as health or finances. Another popular life satisfaction measure is the Life Satisfaction Index (LSI), which covers general feelings of well-being among older people to identify successful aging (7), but the LSI does not assess overall life satisfaction in older people.

Therefore, based on previous studies, we developed a new life satisfaction scale (LSS) to assess the association of health, social factors, and interpersonal relationships with overall satisfaction. The goal of the study was to verify the construct and predictive validities of the LSS to predict depressive symptoms in older adults.

## Materials and Methods

### Participants

The initial sample of participants comprised 4,167 individuals enrolled in the National Center for Geriatrics and Gerontology-Study of Geriatric Syndromes (NCGG-SGS), a Japanese national cohort study (8). Inclusion criteria were age 60 years or older at the time of the baseline survey (September 2015 to February 2017) and residence in Takahama city, a residential suburb of Nagoya.

Based on previous studies that reported certain conditions that could lead to depressive symptoms (9), we excluded participants with a history of stroke (*n* = 240), Parkinson’s disease (*n* = 16), dementia (*n* = 6), and functional decline in basic activities of daily living (*n* = 16), as well as participants with Mini-Mental State Examination (MMSE) (10) scores < 18 (*n* = 14) and those with certified long-term care insurance (*n* = 45). Cases with missing data on risk factors or LSS score (*n* = 98) and on depression (*n* = 101) or depressive symptoms (*n* = 517) at baseline also were excluded. Of the 4,167 initial participants, 1,053 were excluded based on these criteria.

A follow-up mail survey of the 3,114 who completed the baseline survey was conducted an average 30 months after the baseline survey to assess depressive symptoms (July 2018 through September 2018). Of those, 1,322 did not return a completed questionnaire, and the analysis was performed on the data provided by the remaining 1,792 respondents (58% follow-up response rate) (mean age 70.1 ± 6.3 years, 46.4% male). The Ethics Committee of the National Center for Gerontology and Geriatrics approved the study protocol, and informed consent was obtained from all participants before they participated (No. 861-3).

### Variables

#### Depressive Symptoms

Self-report screening tools to identify depressive symptoms were deemed suitable for this community-based study. The 15-item version of the Geriatric Depression Scale (GDS-15) has been validated to screen for depressive symptoms in older people (11, 12). Higher scores indicate more depressive symptoms. A cutoff score of 5 on the GDS-15 has a pooled sensitivity of 88% and specificity of 64%, and a cutoff score of six has a pooled sensitivity of 79% and specificity of 77% for diagnosing depression in older people (13). In the present study, the participants were followed for depressive symptoms using the GDS-15 based on a cutoff value of 5–6 to indicate clinically critical depressive symptoms. The follow-up survey was conducted by mail.

#### Life Satisfaction

A previous study (14–17) developed specific items for each domain of life satisfaction. Based on this, the following life satisfaction domains were selected for the present study: material well-being, health, productivity, intimacy, safety, community, and spirituality or religiosity. We excluded items related to religion and those likely to result in biased responses due to gender differences or family factors (e.g., satisfaction with marriage). We included the general items of each domain and psychological health. Finally, we developed 13 items across five domains: (1) health, (2) community environment, (3) interpersonal relationships, (4) society, and (5) social roles. The 13-item questionnaire intending to capture those domains comprised the following questions. The response options on all of the questions were: 1 = *poor*, 2 = *not very good*, 3 = *good*, and 4 = *excellent.* For the analysis, a total satisfaction score was calculated by summing the individual scores (range = 13–52), and higher scores indicated higher overall satisfaction.

##### Health

Health satisfaction was measured using two global questions: (1) “How satisfied are you with your mental health?” and (2) “How satisfied are you with your physical health?”

##### Community

Community environment satisfaction was measured using responses to three items: (3) “How satisfied are you with your housing?” (4) “How satisfied are you with your community environment or neighborhood?” and (5) “How satisfied are you with your household finances?”

##### Relationships

Interpersonal relationship satisfaction was measured as follows: (6) “How satisfied are you with your relationships with family members?” (7) “How satisfied are you with your relationships with friends?” and (8) “How satisfied are you with your relationships with neighbors?”

##### Society

Satisfaction with Japanese society was measured using responses to two items: (9) “How satisfied are you with Japanese social security, such as pensions and insurance?” and (10) “How satisfied are you with Japanese politics?”

##### Social Roles

Satisfaction with social roles was measured using responses to the following three questions: (11) “How satisfied are you with your social role?” (12) “How satisfied are you with your accomplishments?” and (13) “How satisfied are you with the amount of free time you have for yourself outside of work or household chores?” All of the participants completed in-person interviews that included medical histories taken by licensed, trained nurses.

#### Control Variables

Based on previous studies, the influences of the four demographic variables (age, gender, educational attainment, and household status) and ten health-related variables (six diseases, self-rated health, and three measures of health status) were controlled for in the analysis to adjust for the effects of factors related to depression or depressive symptoms (9, 18, 19) (**Table 1**). The Mini-Mental State Examination (MMSE) often is used to assess the extent of cognitive problems and a frequent component of dementia diagnoses (10). Medical histories included heart disease, pulmonary disease, hypertension, hyperlipidemia, diabetes mellitus, and osteoarthritis of the knee. The responses to items on self-rated health status were on a four-point Likert-type scale, which were dichotomized as “good” or “poor.”

**Table 1:** Comparisons of potential confounders and life satisfaction between the participants with and without depressive symptoms.

Variable	Depressive symptoms		*p*-value
	No (*n* = 1,545)	Yes (*n* = 247)	
	Mean (SD) or *n* (%)	Mean (SD) or *n* (%)	
**Demographic characteristics**			
Age (in years)	69.9 ± 6.2	71.0 ± 6.6	0.017
Gender (ref = male)	714 (46.2)	118 (47.8)	0.648
Education (in years)	11.8 ± 2.4	11.3 ± 2.5	0.006
Household status: living alone (ref = yes)	177 (11.5)	34 (13.8)	0.296
**Health factors**			
Heart disease (ref = yes)	222 (14.4)	38 (15.4)	0.674
Pulmonary disease (ref = yes)	120 (7.8)	29 (11.7)	0.036
Hypertension (ref = yes)	668 (43.2)	121 (49.0)	0.091
Hyperlipidemia (ref = yes)	447 (28.9)	82 (33.2)	0.172
Diabetes mellitus (ref = yes)	178 (11.5)	49 (19.8)	<0.001
Osteoarthritis of the knee (ref = yes)	259 (16.8)	46 (18.6)	0.47
Self-rated health (ref = poor)	159 (10.3)	56 (22.7)	<0.001
Medication (n)	2.3 ± 2.3	3.0 ± 2.8	<0.001
Sleeping pills (ref = yes)	187 (12.1)	38 (15.4)	0.148
Mini-Mental State Examination (score)	27.8 ± 2.2	27.5 ± 2.3	0.069
**Life satisfaction items**			
1) Mental health (ref = poor)	114 (7.4)	73 (29.6)	<0.001
2) Physical health (ref = poor)	192 (12.4)	75 (30.4)	<0.001
3) Housing (ref = poor)	83 (5.4)	23 (9.3)	0.015
4) Community environment (ref = poor)	115 (7.4)	55 (22.3)	<0.001
5) Household finances (ref = poor)	185 (12.0)	73 (29.6)	<0.001
6) Family members (ref = poor)	79 (5.1)	38 (15.4)	<0.001
7) Friends (ref = poor)	96 (6.2)	62 (25.1)	<0.001
8) Neighbors (ref = poor)	179 (11.6)	68 (27.5)	< 0.001
9) Japanese social security (ref = poor)	774 (50.1)	171 (69.2)	<0.001
10) Japanese politics (ref = poor)	816 (52.8)	166 (67.2)	<0.001
11) Social role (ref = poor)	41 (2.7)	32 (13.0)	<0.001
12) Sense of accomplishment (ref = poor)	207 (13.4)	92 (37.2)	<0.001
13) Free time (ref = poor)	77 (5.0)	34 (13.8)	<0.001

### Statistical Analysis

First, those who participated at baseline and returned completed questionnaires at follow-up were divided into two groups: without depressive symptoms (GDS-15 score ≤ 5) and with depressive symptoms (GDS-15 score 6 or higher). The differences between the groups regarding the control variables and life satisfaction were tested using independent samples *t*-tests or Chi-Squared for contingency tests depending on the variable structure.

Construct validity was tested as follows. First, the appropriateness of the data was verified using Bartlett’s Test of Sphericity to ascertain the extent of dependence between items and determine whether the variables were correlated in the sample. Then, following Diener and colleagues (1985), an exploratory factor analysis using Promax orthogonal rotation. Second, to identify the optimal number of extracted factors, a graphic representation of the eigenvalues and the numbers of factors was generated using a scree plot (20). We required that at least 10% of the variance be explained by a retained factor and that at least 60% of the cumulative variance be explained by the set of retained factors (21). The Kaiser-Meyer-Olkin (KMO) test measured the adequacy of the sample. A high internal consistency was anticipated as well as a one-dimensional structure result of the factor analysis. A KMO value greater than 0.7 was used to indicate the adequacy of the data. Instrument reliability was assessed using Cronbach’s alpha coefficient and item-test correlations.

A logistic regression analysis was used to predict the associations between the 13 LSS items and the odds of having depressive symptoms using the binary indicator of depressive symptoms (without v. with depressive symptoms). The statistically significant independent variables were included in the multivariate model. There were three steps: Model 1 controlled for the effects of age, gender, and educational attainment; Model 2 controlled for the effects of variables that indicated significant differences between groups without and with depressive symptoms at baseline; and Model 3 included all the control variables. Late-onset depression and cognitive impairment are closely associated, and cognitive impairment may be a precursor to the onset of depression (22). Thus, we repeated the logistic regression analysis (Model 3) to include the participants with normal cognitive functioning (MMSE scores ≥ 24, n = 1,703).

Receiver operating characteristic (ROC) curves were plotted to determine the LSS cutpoints that best discriminated between the group with depressive symptoms and the group without depressive symptoms. The area under the ROC curve was calculated, and cutpoints for maximizing the sensitivity and specificity of each test were determined using the Youden index (Youden’s J; (23). Based on those cutpoints, the participants were categorized as (1) high life satisfaction or (2) low life satisfaction. The partially and fully adjusted logistic regression models were used to analyze the associations between the LSS cutpoint categories and the depressive state binary indicator. The relative risk of depressive symptoms in the low life satisfaction group was calculated and compared to that of the high life satisfaction group. All statistical tests were considered significant at *p* < 0.05, and all statistical analyses were performed using IBM SPSS Statistics 24.0 (SPSS Inc., Tokyo, Japan).

## Results

### Descriptive Statistics

About 13.8% (*n* = 247) of the 1,792 participants who completed both assessments had depressive symptoms at the follow-up assessment. The overall incidence of depressive symptoms was 55.1 (95% confidence interval (95% CI): 48.7–62.5) per 1,000 person-years. They were significantly different from those without depressive symptoms (*n* = 1,545) regarding age (they were older; *p* = 0.017) and education (they were less educated; *p* = 0.006). Those with depressive symptoms took more medications (*p* < 0.001), and they were more likely to have poor self-rated health (*p* <.001), pulmonary disease (*p* = 0.036), and diabetes (*p* < 0.001) than those without depressive symptoms. The differences between the two groups on all 13 individual LSS items were statistically significant and are shown in **Table 1** above.

### Exploratory Factor Analysis

Bartlett’s Test of Sphericity results indicated high interdependence among the items (Chi-squared = 7,464.2; *p* < 0.001). The KMO value was 0.89, which indicated that the data were adequate for factor analysis. The scree plot depicted an abrupt break or discontinuity before Factor 3, suggesting that only the first two factors had enough meaning to be retained. However, a two-factor solution was not supported by the percentage of variance explained by each of the two factors. Factor 1 explained about 39% of the variance, and Factor 2 explained an additional 10%. We required that at least 10% of the variance be explained by a retained factor and at least 60% of the cumulative variance be explained by the set of retained factors; thus, we selected a four-factor solution. The four-factor construct satisfactorily explained 63% of the total variance.

The rotated pattern of pattern loadings using the Promax rotation of four factors is shown in **Table 2**. Factor 1 made a large and unique contribution to the variance regarding housing, household finances, family members, friends, social roles, accomplishments, and free time. Because these items conceptually related to personal characteristics, Factor 1 was named Personal Satisfaction. Factor 2’s unique and noticeable contributions to the variance were regarding Japanese social security and Japanese politics, and it was named Societal Satisfaction. Factor 3 made a unique and noticeable contribution to the variance through the items on neighbors and the community. Because these items relate to the community environment, Factor 3 was named Community Satisfaction. Factor 4 made a unique and noticeable contribution to the variance regarding mental health and physical health, and it was named Health Satisfaction. The inter-factor correlations of 0.42–0.73 were moderate and positive.

**Table 2:** Exploratory factor analysis results of the 13 LSS items.

Item number and name	Factor 1		Factor 2	Factor 3	Factor 4
3. Housing		0.71	0	−0.02	−0.11
5. Household finances		0.48	0.20	−0.09	0.10
6. Family members		0.67	−0.05	0.11	−0.11
7. Friends		0.43	−0.08	0.26	0.08
11. Social role		0.63	−0.07	0.02	0.16
12. Accomplishment		0.42	0.15	−0.04	0.21
13. Free time		0.57	−0.04	−0.02	0.01
9. Japanese social security		0.02	0.90	−0.04	−0.04
10. Japanese politics		−0.07	0.67	0.12	0.02
4. Community environment		−0.01	0.06	0.74	0.05
8. Neighbors		0.05	0.03	0.74	−0.03
1. Mental health		−0.04	−0.04	−0.03	0.90
2. Physical health		0.01	0.01	0.08	0.48

### Internal Reliability

The internal reliability of the LSS was assessed using Cronbach’s alpha coefficient, which was 0.86 in the sample. The inter-item correlations were moderate to high for the scale (item-test), and the coefficients ranged from 0.42 to 0.63 (**Table 3**).

**Table 3:** Pearson’s bivariate correlations (*r*) matrix, inter-total correlations, and Cronbach’s alpha coefficients for the LSS items.

LSS item	1	2	3	4	5	6	7	8	9	10	11	12	13	ITC^a^	α^b^
1. Mental health														0.58	0.850
2. Physical health	0.45													0.45	0.858
3. Housing	0.31	0.25												0.51	0.854
4. Community environment	0.34	0.31	0.35											0.60	0.849
5. Household finances	0.36	0.33	0.40	0.32										0.55	0.852
6. Family members	0.34	0.24	0.45	0.37	0.32									0.55	0.852
7. Friends	0.39	0.29	0.32	0.45	0.33	0.43								0.57	0.850
8. Neighbors	0.31	0.26	0.32	0.6	0.31	0.40	0.43							0.56	0.851
9. Japanese social security	0.27	0.21	0.18	0.31	0.35	0.18	0.21	0.27						0.44	0.859
10. Japanese politics	0.25	0.2	0.17	0.31	0.27	0.19	0.19	0.29	0.61					0.42	0.860
11. Social role	0.48	0.34	0.40	0.40	0.41	0.48	0.49	0.38	0.22	0.22				0.63	0.848
12. Accomplishment	0.48	0.28	0.37	0.35	0.41	0.38	0.38	0.35	0.33	0.29	0.44			0.59	0.849
13. Free time	0.31	0.21	0.33	0.28	0.28	0.32	0.39	0.28	0.18	0.14	0.44	0.32		0.46	0.857

a Item-Total Correlation.

b Cronbach’s reliability coefficient alpha.

### Logistic Regression Results

The logistic regression analysis predicted the effects of the individual LSS items and whether a participant was in the depressive symptom group (**Table 4**). In the partially adjusted estimation (Model 1), the likelihood of having depressive symptoms was higher for those with poor satisfaction on all 13 items. Similar ORs were estimated in Model 2 and 3.

**Table 4:** The predictive influences of the 13 LSS items on depressive symptoms; logistic regression odds ratios (ORs) with 95% Confidence Intervals (CI).

Variable (LSS item)	Model 1^a^		Model 2^b^		Model 3^c^	
	Odds Ratio (95% CI)	*p*-value	Odds Ratio (95% CI)	*p*-value	Odds Ratio (95% CI)	*p*-value
3. Housing	1.85 (1.14–3.01)	0.013	1.71 (1.05–2.81)	0.030	1.68 (1.03–2.77)	0.04
6. Family members	3.52 (2.31–5.34)	<0.001	3.52 (2.30–5.38)	<0.001	3.48 (2.27–5.32)	<0.001
11. Social role	5.78 (3.54–9.42)	<0.001	5.39 (3.27–8.91)	<0.001	5.41 (3.27–8.97)	<0.001
13. Free time	3.49 (2.25–5.41)	<0.001	3.29 (2.10–5.15)	<0.001	3.42 (2.18–5.37)	<0.001
5. Household finances	3.10 (2.26–4.26)	<0.001	2.86 (2.08–3.95)	<0.001	2.84 (2.05–3.94)	<0.001
7. Friends	5.42 (3.77–7.79)	<0.001	4.93 (3.41–7.12)	<0.001	4.94 (3.41–7.17)	<0.001
12. Accomplishment	4.02 (2.98–5.44)	<0.001	3.84 (2.82–5.22)	<0.001	3.83 (2.82–5.22)	<0.001
9. Japanese social security	2.41 (1.79–3.24)	<0.001	2.30 (1.71–3.11)	<0.001	2.31 (1.71–3.11)	<0.001
10. Japanese politics	1.92 (1.44–2.57)	<0.001	1.84 (1.37–2.46)	<0.001	1.86 (1.39–2.49)	<0.001
8. Neighbors	3.08 (2.22–4.26)	<0.001	2.96 (2.13–4.11)	<0.001	2.96 (2.12–4.12)	<0.001
4. Community environment	3.60 (2.52–5.16)	<0.001	3.39 (2.36–4.88)	<0.001	3.36 (2.33–4.85)	<0.001
1. Mental health	5.45 (3.89–7.64)	<0.001	4.65 (3.24–6.68)	<0.001	4.70 (3.26–6.77)	<0.001
2. Physical health	3.11 (2.28–4.25)	<0.001	2.48 (1.69–3.65)	<0.001	2.48 (1.68–3.64)	<0.001

a Model 1 included age, gender, and educational attainment as control variables.

b Model 2 included age, educational attainment, pulmonary disease, diabetes mellitus, self-rated health, and medication as control variables.

c Model 3 included age, gender, educational attainment, household status, and ten health factors as control variables.

We conducted a sensitivity analysis using a logistic regression with the participants having MMSE scores ≥ 24. We found no significant difference for housing, but other LSS items showed similar odds ratios compared to the results for the overall sample (**Table 5**).

**Table 5:** The predictive influence of the 13 LSS items on depressive symptoms in the individuals having MMSE score 24 points and over; logistic regression odds ratios (ORs) with 95% Confidence Intervals (CI).

	Odds Ratio (95% CI)	*p*-value
3. Housing	1.57 (0.94–2.63)	0.09
6. Family members	3.29 (2.13–5.09)	<0.001
11. Social role	6.16 (3.68–10.31)	<0.001
13. Free time	3.76 (2.38–5.95)	<0.001
5. Household finances	2.93 (2.10–4.08)	<0.001
7. Friends	4.86 (3.32–7.10)	<0.001
12. Accomplishment	3.89 (2.83–5.35)	<0.001
9. Japanese social security	2.36 (1.73–3.22)	<0.001
10. Japanese politics	1.84 (1.36–2.49)	<0.001
8. Neighbors	2.92 (2.08–4.12)	<0.001
4. Community environment	3.30 (2.26–4.80)	<0.001
1. Mental health	4.68 (3.21–6.81)	<0.001
2. Physical health	2.64 (1.78–3.94)	<0.001

Model included age, gender, educational attainment, household status, and ten health factors as control variables.

### Cutpoints

During the follow-up period, 247 (13.8%) of the 1,792 participants experienced new depressive symptoms. The ROC of the LSS was 0.74 (95% CI: 0.70–0.77, *p* < 0.001), and the Youden index determined the cutpoints regarding the development of depressive symptoms identified by the LSS as 39 points. The participants were classified as high life satisfaction (60%, *n* = 1,068) or low life satisfaction (40%, *n* = 724). In Model 1 of the logistic regression analysis, the cutpoints (risk v. no risk) using the total life satisfaction score were significantly related to the development of depressive symptoms (OR = 5.47, 95% CI = 4.02–7.44, *p* < 0.001). The fully adjusted model 3 indicated that the LSS was a statistically significant independent predictor of the incidence of depressive symptoms (OR = 5.14, 95% CI = 3.76–7.04, *p* < 0.001). The relative risk of the low life satisfaction group for developing depressive symptoms compared to the high life satisfaction group was 2.39. Similar odds ratios were observed for Model 3 among the participants with MMSE scores ≥ 24 (OR = 5.66, 95% CI = 4.07–7.88, *p* < 0.001).

## Discussion

This study aimed to determine the structural validity and predictive validity of a new instrument to measure life satisfaction, the 13-item LSS, regarding depressive symptoms. Four factors emerged from the exploratory factor analysis: (1) personal satisfaction, (2) societal satisfaction, (3) community satisfaction, and (4) health satisfaction. The scale’s internal consistency for this sample of older Japanese was acceptably reliable (Cronbach’s alpha = 0.86). The four-factor construct satisfactorily explained 63% of the total variance and presented factor weights that varied between 0.42 and 0.90. These results are consistent with factor analysis results found by many previous studies using the SWLS, which explained about 52 to 66% of the variance (24–26). Moreover, the LSS had good internal consistency (Cronbach’s alpha = 0.86), which is similar to the findings of studies using the SWLS in various languages and populations (ranging from 0.79 to 0.89) (27).

In the sample of 1,792 participants who completed the baseline and follow-up assessments, 247 (13.8%) were identified with depressive symptoms and the overall incidence of depressive symptoms was 55.1 per 1,000 person-years. A 3-year observational cohort study identified that the incidence of depression using 6 as the GDS cutoff score was 42.7 (95% CI 38.0–47.9) per 1,000 person-years (28). Another prospective study reported that 8.4% (79/945) of participants were newly classed as depressed (GDS-15 score > 5) at 2-year follow-up (41.8 (95% CI 33.5–52.1) per 1,000 person-years) (29). The participants in our study were slightly more likely than those in these previous studies to develop depressive symptoms. However, a direct comparison with other studies is difficult due to the varying operationalizations of depressive disorders, heterogeneous age groups, and different ways to calculate incidence (30). The Leiden 85-plus Study followed the participants without depression at baseline, defined as having a GDS-15 score of 0–2 points at age 85 years, over a 4-year period. The study identified that the incidence of depression using 5 as the GDS cutoff score was 68 (95% CI 50-85) per 1,000 person-years (31). This high incidence of depression may be due to older participants and different cutoffs.

Using the score cutoff point on total life satisfaction, the depressive symptom incidence rate among those with low satisfaction was 24.9%, which was higher than among those with high satisfaction (6.3%). The logistic regression analyses revealed that the LSS predicted the onset of depressive symptoms even after adjusting for the effects of many confounding factors. Thus, we concluded that the LSS was an independent predictor of the development of depressive symptoms in older adults. The results suggest the need to introduce a questionnaire into the healthcare system to detect early life dissatisfaction that leads to depressive symptoms and help reduce suicide rates from depression.

An important limitation of our study is that the participants were not randomly recruited, which might have led to an underestimation of the prevalence of low life satisfaction and depressive symptoms because the participants were relatively healthy older people who could access health checkups from their homes. Second, 1,322 participants were lost in the follow-up (42%), which might have led to an underestimation of the development of depressive symptoms as a survival effect. However, the participants had a slightly higher incidence of depressive symptoms than those in these previous studies. We considered that the effects of the sampling bias and survival effects were minimal in the participants of this study. Third, the components of life satisfaction are so diverse that not all of them can be explained on LSS. Fourth, the NCGG-SGS is a population survey and therefore includes participants with various characteristics; it is possible that some participants have confounding factors for the development of depressive symptoms. Since we deleted participants with poor health conditions in this study, we cannot apply the results of this study to these older adults. Despite these limitations, the notable strengths of the study are the large cohort size recruited from a single community and adjustment of various confounding factors.

## Conclusions

We created a new life satisfaction scale (LSS) comprised of four factors: personal satisfaction, societal satisfaction, community satisfaction, and health satisfaction to assess the incidence of depressive symptoms. The scale’s internal consistency and predictive validity were high, indicating that the LSS could reliably be used in psychological and geriatric research to identify the risk of depression among older adults. Further research is required to identify the novel candidate biomarkers necessary for the early detection of depression.

## Data Availability Statement

The datasets generated for this study are available for verification of the results in this study on request to the corresponding author.

## Ethics Statement

The studies involving human participants were reviewed and approved by The Ethics Committee of the National Center for Gerontology and Geriatrics. The patients/participants provided their written informed consent to participate in this study. Written informed consent was obtained from the individual(s) for the publication of any potentially identifiable images or data included in this article.

## Author Contributions

HS planned the study, wrote the first draft of the manuscript, and coordinated the review and editing process leading to the final manuscript. SL, SB, and RH suggested many ideas that were pursued in the research and participated in the planning, editorial, and review processes that led to the final manuscript. All authors contributed to the article and approved the submitted version.

## Funding

This work was supported by the Japan Agency for Medical Research and Development (15dk0107003h0003, 15dk020 7004h0203), and Kao Corporation, Japan. The funder Kao Corporation was not involved in the study design, collection, analysis, interpretation of data, the writing of this article or the decision to submit it for publication.

## Conflict of Interest

This study was partly funded by Kao Corporation.
